# Post-pandemic Rise in Mature Cataracts and Delayed Ophthalmic Care: A Retrospective Analysis From a Tertiary Center in Italy

**DOI:** 10.7759/cureus.99060

**Published:** 2025-12-12

**Authors:** Rosario Alfio Umberto Lizzio, Francesco Polimeni, Simone Caboni, Fabrizio D'Ancona, Stefano Mattioli, Paolo Nucci, Stela Vujosevic

**Affiliations:** 1 Eye Clinic, IRCCS MultiMedica, Milan, ITA; 2 Department of Ophthalmology, University of Milan, Milan, ITA; 3 Department of Biomedical, Surgical and Dental Sciences, University of Milan, Milan, ITA

**Keywords:** cataract surgery, coronavirus pandemic, covid – 19, mature cataract, public health

## Abstract

Background and objective

The coronavirus disease 2019 (COVID-19) pandemic led to a worldwide decline in elective medical procedures, including cataract surgeries. This study aimed to assess the incidence of mature cataracts one year after the official end of the pandemic at a tertiary ophthalmology center in Milan, Italy.

Methods

A retrospective analysis was conducted involving patients who underwent cataract surgery at IRCCS MultiMedica Eye Clinic in 2019 and from May 2023 to May 2024. Inclusion criteria encompassed best-corrected visual acuity (BCVA) <20/100 due to mature cataract or surgery via extracapsular cataract extraction (ECCE). Demographic and clinical data were compared across the two periods. Patients from 2023-24 were also surveyed on reasons for delayed care.

Results

The incidence of mature cataracts increased from 10/2681 (0.37%) in 2019 to 27/2660 (1.01%) in 2023-24 (p = 0.0004). The mean patient age was 81.9 ± 5.2 years in 2019 compared with 76.2 ± 8.6 years in 2023-24 (p = 0.057). Sex distribution was comparable between the two periods (5/10 males (50%) vs. 13/27 males (48.1%), p = 1.00). Diabetes prevalence was 4/10 (40%) vs. 12/27 (44.4%) (p = 0.407). ECCE was performed in 4/10 (40%) vs. 13/27 (48.1%) (p = 0.72). Among 2023-24 patients, 17/25 respondents (68.0%) reported prior COVID-19 infection. Reasons for delayed ophthalmic care included fear of infection (10/25, 40.0%), lack of appointment availability (8/25, 32.0%), and underestimation of need (9/25, 36.0%).

Conclusions

A significant increase in mature cataracts was observed one year post-pandemic. These findings describe an observable post-pandemic trend and highlight a relevant public health signal that may inform future planning of ophthalmic services.

## Introduction

The coronavirus disease 2019 (COVID-19) pandemic, caused by the Severe acute respiratory syndrome coronavirus 2 (SARS‑CoV‑2) virus, emerged in late 2019 in Wuhan, China, and rapidly spread worldwide, triggering one of the most significant public health crises in modern history. By March 2020, the World Health Organization (WHO) declared COVID-19 a pandemic, leading to widespread lockdowns, border closures, and extraordinary strains on healthcare systems across the globe [1]. The virus’s spread impacted not only health but also reshaped economies, societies, and daily lives, with Italy being one of the earliest and hardest-hit countries in Europe.

Italy recorded its first COVID-19 cases in January 2020, but the situation escalated dramatically in February, with the Lombardy and Veneto regions experiencing rapid outbreaks. The country soon became the epicenter of the pandemic in Europe. In response, the Italian government enforced strict nationwide lockdowns and healthcare measures, aiming to curb the virus’s transmission. As vaccination campaigns expanded globally and treatments improved, the impact of COVID-19 progressively diminished, resulting in a substantial decline in severe cases and fatalities. By May 2023, the WHO officially declared the end of COVID-19 as a global health emergency, marking a crucial turning point in the pandemic response [2].

Cataract surgery is one of the most commonly performed procedures worldwide, providing substantial improvements in vision and quality of life for patients. Cataracts alone account for 45.4% of global blindness and 38.9% of visual impairment, affecting an estimated 15.2 million blind individuals and 78.8 million people with visual impairment worldwide [3]. In high-income countries, cataract surgery has significantly reduced the prevalence of cataract-related blindness, while in low- and middle-income countries, barriers to access still pose major challenges to widespread treatment. Epidemiologically, cataracts are closely linked to aging, with the majority of cases occurring in people over the age of 60. Lifestyle factors, such as smoking, UV exposure, and diabetes, have been associated with an increased risk of developing cataracts at earlier ages, which highlights the importance of preventative care and screening [4].

Like all ophthalmic procedures [5], cataract surgery also saw a reduction during the COVID-19 pandemic, as extensively documented in the literature. At the end of the pandemic, we noticed a marked increase in mature cataracts, which had been quite rare in our hospital before, as it serves the population residing in the center of Milan, with easy access to healthcare and regular prevention check-up services. The purpose of this study is to retrospectively assess the number of mature cataracts one year after the end of the pandemic at our tertiary care hospital in Milan, one of the Italian cities most heavily affected by the COVID-19 pandemic.

## Materials and methods

The research was conducted at the Eye Clinic, IRCCS MultiMedica in Milan, Italy. The study employed a retrospective review of anonymized medical records of patients who underwent cataract surgery at IRCCS MultiMedica. Because no identifiable data were used and no intervention was performed, the requirement for formal ethical approval was waived in accordance with institutional and national regulations. All procedures adhered to the principles of the Declaration of Helsinki.

We retrospectively reviewed electronic medical records of all patients who underwent cataract surgery between January and December 2019 and between May 2023 and May 2024, one year after the official end of the pandemic. Data extraction was performed by two investigators (FP, SC), using electronic charts only. All eligible records were extracted and contained complete information for the variables analyzed (age, sex, best-corrected visual acuity (BCVA), comorbidities, and surgical technique), with no missing data; therefore, no imputation was required. Inclusion criteria consisted of a mature cataract confirmed by Lens Opacities Classification System III (LOCS III) or an indication for extracapsular cataract extraction (ECCE) due to a dense nucleus, with a BCVA <20/100 attributable exclusively to the cataract. Exclusion criteria included the presence of macular or corneal disease, traumatic cataract, or incomplete medical records.

Throughout both study periods (2019 and 2023-24), the indication for ECCE versus phacoemulsification was based on lens density, red reflex quality, and expected phacoemulsification energy requirements. Surgical protocols, surgical staff, and phacoemulsification machine availability remained unchanged throughout the study period. ECCE was therefore performed only when nuclear hardness or poor visibility made phacoemulsification unsuitable. Patients from both 2019 and 2023-24 were contacted by telephone to obtain verbal consent for participation in the study. In addition, patients from the 2023-24 group were surveyed regarding the reasons for the delay in cataract surgery. The survey was exploratory in nature and not derived from a previously validated questionnaire.

Statistical analysis

Statistical analyses were performed using GraphPad Prism version 10.4.0 (GraphPad Software, Boston, MA). Normality of continuous variables was assessed using the Shapiro-Wilk test. Parametric testing (t-test) was used for normally distributed data, while non-parametric categorical comparisons were analyzed using Fisher's exact test. Categorical variables were expressed as proportions with corresponding 95% confidence intervals (95% CI, Wilson method). Differences in proportions between groups were analyzed using the chi-square test; for all categorical comparisons with small expected cell counts, Fisher’s exact test was applied. For each chi-square analysis, the chi-square statistic (χ²), degrees of freedom (df), p-value, and effect size (phi coefficient, φ) were reported. Effect sizes were interpreted according to conventional thresholds (φ = 0.10 small, 0.30 medium, 0.50 large). A two-sided p<0.05 was considered statistically significant.

## Results

In 2019, 10 cases of mature cataract (10/2,681, 0.37%) were recorded, compared with 27 cases (27/2,660, 1.01%) in 2023-24, representing a statistically significant increase (χ²(1) = 12.39, p = 0.0004, φ = 0.048). The mean age was slightly lower in 2023-24 (76.2 ± 8.6 years) than in 2019 (81.9 ± 5.2 years), though this difference was not statistically significant (p = 0.057). Sex distribution was similar between periods (5/10 males, 50% vs. 13/27, 48.1%; p = 1.00). Diabetes prevalence did not differ significantly (4/10, 40% vs. 12/27, 44.4%; p = 0.407). ECCE was performed in 4/10 patients (40%) in 2019 vs. 13/27 (48.1%) in 2023-24 (p=0.72). Visual impairment (BCVA <20/400) occurred in 6/10 (60%) in 2019 and 24/27 (88.9%) in 2023-24, showing a worsening trend, albeit without statistical significance (p = 0.15).

The telephone survey conducted among patients from 2023-24 achieved a 92.6% response rate (25/27 patients). Of the respondents, 17/25 (68.0%) reported a prior SARS-CoV-2 infection. Reported reasons for delayed ophthalmic care included fear of infection (10/25, 40.0%), limited appointment availability (8/25, 32.0%), and underestimation of visual decline (9/25, 36.0%). Recall bias cannot be excluded (Table 1).

**Table 1:** Comparison of demographic and clinical characteristics of patients with mature cataract in 2019 vs. 2023-2024 CI: confidence interval; SD: standard deviation; BCVA: best-corrected visual acuity; ECCE: extracapsular cataract extraction

	Year 2019	Year 2023-24	P-value	Effect size (φ)
Mature cataract, n (%, 95% CI)	10 (0.37%, 0.20–0.69)	27 (1.01%, 0.69–1.47)	0.0004	0.048
Male/female (male %)	5/5 (50%)	13/14(48.1%)	1.00	0.007
Age, years, mean ± SD	81.90 ± 5.24	76.2 ± 8.62	0.0570	—
Diabetes, n (%)	4 (40.0%)	12 (44.4%)	0.407	0.03
BCVA <20/400, n (%)	6 (60.0%)	24 (88.9%)	0.15	0.25
ECCE, n (%)	4 (40.0%)	13 (48.1%)	0.72	0.08

## Discussion

Cataract is the leading cause of blindness and the second leading cause of visual impairment worldwide, after uncorrected refractive errors. It accounts for 45.4% of global blindness and 38.9% of visual impairment, affecting approximately 15.2 million blind individuals and 78.8 million people with visual impairment globally [3]. The prevalence of cataracts is 1232.33 individuals per 100,000 individuals, and it is expected to remain relatively stable by the year 2050. Cataract surgery has been recognized as one of the most frequently performed surgical procedures in recent decades [5]. In 2019, the emergence of the COVID-19 pandemic created a global health crisis, prompting many healthcare systems to prioritize care for affected individuals, often at the expense of other medical procedures. This underscores the importance of evaluating the impact of COVID-19 on healthcare services during this period.

Lim et al. [6] reported a decline in cataract surgeries in Malaysia during the COVID-19 pandemic. Similarly, Rossi et al. [7] described a decrease in Brazil in 2020, followed by a subsequent rise in procedures. Magalhaes et al. [8] revealed that cataract surgeries in Brazil dropped by around 50% during the COVID-19 period. This trend was also observed in more robust healthcare systems. Zhou et al. [9] presented global data showing major decline even in strong economies: 26.38% in China, 10.58% in the United States, 20.49% in Germany, 27.97% in Spain, and 22.64% in Italy. In 2021, dell’Omo et al. reported a reduction of cataract surgeries by 97.7-97.8% [10]. Toghyani et al. [11] observed an increase in waiting times and reduced operating room availability, contributing to fewer surgeries. These findings are consistent with several studies pointing to a significant drop in cataract surgeries during the pandemic [12,13]. Post-pandemic, there has been a notable rebound in the number of cataract surgeries. This indicates that the pandemic’s impact on healthcare services, though considerable, was temporary [5].

In this study, we analyzed the increase in mature cataracts one year after the official end of the pandemic. In 2019, mature cataracts represented 10/2681 surgeries (0.37%), compared with 27/2660 (1.01%) in 2023-24, showing a statistically significant rise despite the small absolute numbers. However, although our findings demonstrate a clear increase, the retrospective design does not allow us to establish a causal relationship with COVID-19. In the absence of longitudinal trend analysis or regression modeling, these results should be viewed as a descriptive comparison between the two periods rather than evidence of association or causation. To our knowledge, this study is among the first to quantify the post-pandemic incidence of mature cataracts in a European tertiary care setting.

Data on the prevalence of mature cataracts in high-income countries remain limited. In the UK, Sim et al. reported 52,519 brunescent, white, or mature cataracts among 908,689 cataract surgeries over six years (5.78%) [14], yet without a specific pandemic-focused comparison. Bhalerao et al. observed a marked rise in India, with mature cataracts increasing from 194/1298 (15%) in 2019 to 191/455 (42%) in 2020 (p<0.0001) [15]. Unlike studies conducted during the pandemic surge phase, our analysis focused on the period one year after full service resumption, when the long-term consequences of delayed care became more evident. This difference in timing and geographical context highlights how our findings add complementary evidence to the existing literature, particularly from a European perspective, where comparable post-pandemic analyses are currently lacking.

Vedachalam et al. [16] investigated reasons for delaying cataract surgery during the pandemic. Similarly, we found that fear of infection, lack of appointment availability, and underestimation of visual decline were the main factors reported by our patients. Nonetheless, part of the observed increase may also reflect unrelated factors - for example, natural year-to-year fluctuations in case mix or temporary changes in referral pathways. These points should be interpreted as hypothetical alternative explanations rather than proven causal factors. Although we cannot exclude their possible influence, they may have contributed in addition to pandemic-related delays. The high impact of COVID-19 in Lombardy may also have contributed to behavioral changes such as increased fear of infection.

Regarding age, we observed a nonsignificant trend toward younger patients presenting with mature cataracts after the pandemic, a pattern also seen in Indian and Asian populations [5,15]. The average age in our cohort is consistent with Northern European data [14,17], although these reports do not specifically analyze mature cataracts. In the cited databases, the mean age of cataract surgery was reported as 74.5 ± 9.8 years in the UK [14] and 74.4 years in Sweden [17], values comparable to our post-pandemic cohort (76.2 ± 8.6 years). Gender distribution, severity of visual impairment, surgical technique, and diabetes prevalence did not differ significantly across the study periods. These findings suggest that the main difference between the years was the number of patients arriving with advanced lens opacities, not their underlying clinical profile. Also, the slight increase in ECCE procedures in 2023-24 (48.1% vs. 40%) should be interpreted as reflective of cataract density, since surgical indications and workflow for ECCE did not change across study periods.

Limitations and strengths

This study has several limitations. Firstly, its retrospective design, relatively small sample size, and single-center setting may limit the generalizability of the findings. The lack of detailed longitudinal data prevents a more robust trend evaluation over time, and patient-reported reasons for delayed care may be subject to recall bias. Importantly, although a significant rise in mature cataracts was observed, we did not perform regression modelling or causal-inference/time-series analyses; therefore, our findings reflect a descriptive comparison rather than a causal relationship with the COVID-19 pandemic. The absence of statistical significance in some comparisons may also reflect limited statistical power due to the small sample size in each period. Future multicenter studies with larger cohorts and longer follow-up are required to validate and expand upon these observations.

Although our findings are not particularly novel and largely reflect an expected consequence of the COVID-19 pandemic, they provide quantitative evidence of a clinically and socially relevant issue: the persistence of mature cataracts even after the official end of the pandemic. This observation shows that the disruption of elective ophthalmic care produced long-lasting effects even in a high-income setting with traditionally easy access to services. Patients presented to surgery with more advanced visual impairment, increasing surgical complexity, and the risk of complications, imposing a greater functional and socioeconomic burden. These results emphasize the need for resilient healthcare planning capable of preserving access to essential ophthalmic services during future crises.

## Conclusions

This study found a statistically significant increase in mature cataracts one year after the COVID-19 pandemic. While causality cannot be inferred, this trend emerging in a highly accessible healthcare setting suggests that pandemic-related service disruption may have contributed to delayed presentation. These results highlight the need to strengthen continuity of essential ophthalmic care during future health emergencies.
